# Response to: “Before blaming a COVID vaccine for cytotoxic lesions of the corpus callosum all other differentials must be ruled out”

**DOI:** 10.1007/s00234-022-03057-x

**Published:** 2022-09-23

**Authors:** Hiroya Ohara, Hironori Shimizu, Takehito Kasamatsu, Akihiro Kajita, Kenji Uno, Khin Wee Lai, Balachandar Vellingiri, Kazuma Sugie, Masako Kinoshita

**Affiliations:** 1 Department of Neurology, Minaminara General Medical Center, Yoshino, Nara, Japan; 2 Department of Neurology, Nara Medical University School of Medicine, Kashihara, Nara, Japan; 3 Department of Infection, Minaminara General Medical Center, Yoshino, Nara, Japan; 4 Department of Biomedical Engineering, Faculty of Engineering, University of Malaya, Kuala Lumpur, Malaysia; 5 Department of Human Genetics and Molecular Biology, Bharathiar University, Coimbatore, Tamil Nadu India; 6 Department of Neurology, National Hospital Organization Utano National Hospital, 8 Ondoyama-cho, Narutaki, Ukyo-ku, Kyoto, 616-8255 Japan

To the Editors,

We greatly appreciate the comments by Dr. Finsterer on our previous article describing patients who showed cytotoxic lesions of the corpus callosum (CLOCCs) after coronavirus disease 2019 (COVID-19) messenger ribonucleic acid (mRNA) vaccination, which provided us an opportunity to describe diagnostic strategies for neurological symptoms and to provide further information on Patient 2 [1, 2].

Regarding the first criticism on our description that side effects of severe acute respiratory syndrome coronavirus 2 (SARS-CoV-2) vaccinations are not life-threatening, we would like to clarify that this notion is derived from previous studies which evaluated neurological adverse events among healthcare workers after COVID-19 mRNA vaccination [3, 4]. The most common neurological symptoms were headache, dizziness, decreased appetite, muscle spasm, decreased sleep quality, and brain fogging [3, 4]. Nobody doubts that these symptoms are usually considered mild. However, what we emphasized in our article was the importance of early detection of potentially serious neurological manifestations and require adequate therapy as soon as possible, in concordance with the concern by Dr. Finsterer that systemic immunological and vascular adverse reactions to SARS-CoV-2 vaccinations can be fatal [1].

As for the second criticism on insufficient investigation of Patient 2, we apologize for providing wrong information in the previous manuscript [2], and would like to discuss differential diagnosis based on additional clinical data. Examination of the cerebrospinal fluid (CSF) underwent on day 17 and the result was unremarkable (0.7 cells/µL (monocyte count 0.7 cells/µL, polycyte count 0 cells/µL), normal ≤ 5 cells/µL; protein 25 mg/mL, normal 10–40 mg/mL; immunoglobulin G index 0.49, normal < 0.73; and negative oligoclonal band). Blood coagulation test on the same day revealed that the international normalized ratio was 1.09 (normal 0.85–1.15), activated partial thromboplastin time 30.7 s (normal 24.3–36.0 s), and fibrin degradation products 2.0 µg/mL (normal < 4.0 µg/mL). Magnetic resonance angiography of the brain was also unremarkable on day 17. Thus, focal encephalitis, vasculitis, venous sinus thrombosis, and systemic autoimmune disorders are unlikely. In addition, no abnormality was shown by ophthalmologic observation of anterior ocular segment and of the fundus on day 22. These findings can exclude possibilities of multiple sclerosis, neuromyelitis optica, and other systemic disorders causing retinopathy, vasculitis, and uveitis. MRI of the spinal cord was not performed because neurological examination did not indicate myelitis; dysesthesia distributed in distal part of all limbs, deep tendon reflexes were normal, abnormal reflexes including Babinski signs were negative, and there was no urinary or bowel dysfunction. Considering the full improvement of splenial lesion in MRI on day 29 [2], and lack of abnormality in perfusion-weighted imaging 2 months later, ischemic stroke and atherosclerosis are quite unlikely. Marchiafava-Bignami disease (MBD) should be considered in patients with chronic alcohol abuse. In its acute form, the genu and splenium of the corpus callosum are commonly involved, and MRI shows T1-weighted hypointensity, T2-weighted and fluid-attenuated inversion recovery image hyperintensity, and often with restricted diffusion within the corpus callosum [5]. In contrast, our Patient 2 was an occasional alcohol drinker, and her blood test showed no sign of hepatic dysfunction or malnutrition. Serum vitamin B_1_ was 39 ng/mL (normal 24–66 ng/mL) and vitamin B_12_ was 218 pg/mL (normal 180–914 pg/mL) on day 17. CLOCCs are defined as highly restricted ovoid lesions in the midline of the splenium as shown in our patients [2], whereas lesions of the corpus callosum in patients with MBD widely distribute with blurred margin, and thus are usually not described as CLOCCs. Diagnosis of acute disseminated encephalomyelitis (ADEM) require pleocytosis in the CSF. Lesions of ADEM typically reside in the deep and subcortical white matter and can occur in the thalamus and basal ganglia. We would like to remind that contrast-enhanced MRI is useful to evaluate ADEM but sole involvement of corpus callosum is rare [2]. General physical examination of Patient 2 was not suggestive of influenza and other viral infections; the patient was afebrile and there was no sign of inflammation of the upper respiratory tract. Though, in immunocompromised hosts, it is recommended to investigate possibility of infections and to consider antibacterial, antiviral, and antimycotic prophylaxis before starting immunotherapy.

Admitting that precise diagnostic procedure is crucial and therapeutic intervention should be based on the most plausible diagnosis, full establishment of diagnosis requires at least several weeks, causes delay in treatment, and directly affects prognosis of patients in clinical practice. Correlation between radiological characteristics of lesions involving the corpus callosum including CLOCCs and various neurological disorders will enable us to establish diagnostic and therapeutic strategies from etiological point of view.

## Data Availability

The data that support the findings of this study are available on request from the first author [H.O.]. The data are not publicly available due to information that could compromise research participant privacy.

## Abbreviations

CLOCCs: Cytotoxic lesions of the corpus callosum
COVID-19: Coronavirus disease 2019
mRNA: Messenger ribonucleic acid
SARS-CoV-2: Severe acute respiratory syndrome coronavirus 2
MRI: Magnetic resonance imaging
CSF: Cerebrospinal fluid
MBD: Marchiafava-Bignami disease
ADEM: Acute disseminated encephalomyelitis
